# Incidence and risk factors for psychological distress in adult female patients with breast cancer: a systematic review and meta-analysis

**DOI:** 10.3389/fpsyt.2024.1309702

**Published:** 2024-03-13

**Authors:** Lin Tao, Yuping Xiang, Xiaohong Zeng, Lan Fu, Junying Li, Hong Chen

**Affiliations:** ^1^ Cancer Day-Care Unit, Division of Medical Oncology, Cancer Center, West China Hospital, Sichuan University/West China School of Nursing, Sichuan University, Chengdu, China; ^2^ Department of Critical Care Medicine, West China Hospital, Sichuan University/West China School of Nursing, Sichuan University, Chengdu, China; ^3^ Thoracic Oncology Ward, Cancer Center, West China Hospital, Sichuan University/West China School of Nursing, Sichuan University, Chengdu, China; ^4^ Department of Nursing, West China Hospital, Sichuan University/West China School of Nursing, Sichuan University, Chengdu, China

**Keywords:** breast cancer, psychological distress, incidence, risk factor, review

## Abstract

**Introduction:**

Cancer-related distress can be described as a complex and unpleasant combination of psychological (such as cognitive, behavioral, and emotional), social, and spiritual challenges that may impact an individual’s ability to effectively cope with the physical symptoms of cancer and its treatment. Existing literature has confirmed psychological distress (PD) as an important sequela of breast cancer diagnosis and treatment. However, the incidence and risk factors for PD in adult female patients with breast cancer remain unclear; therefore, focusing on the PD of female breast cancer patients is meaningful, as they are at highest risk of contracting breast cancer, and might differ in their coping styles from men.

**Objective:**

This review aimed to identify the incidence and risk factors for PD in adult woman patients with breast cancer, and to help guide targeted intervention to prevent distress.

**Method:**

PubMed, Embase, Cochrane Library, CINAL, PsycINFO, China Knowledge Resource Integrated Database, Wanfang Database, the Chinese Biomedical Database, and Weipu Database were searched for data regarding the incidence and risk factors of PD in adult women with breast cancer.

**Results:**

The prevalence of PD, assessed using the distress thermometer, ranged between 11.2%–86.7%, and a meta-analysis of 47 studies with 15,157 adult female breast cancer patients showed that the pooled prevalence was 52.0%. Further, this study identified 40 risk factors. However, owing to the inclusion of at least two studies for a certain risk factor, 10 risk factors were merged for the meta-analysis. Independent risk factors included higher education level, late-stage tumor, emotional concerns, no medical insurance, modified radical mastectomy, and history of depression; age and neuroticism were not associated with PD; and higher monthly income was revealed as a protective factor against it.

**Conclusion:**

The incidence of PD in female patients with breast cancer is high and it involves 10 risk factors, though some are controversial owing to insufficient evidence. Further research is needed to explore the underlying mechanisms of PD and develop risk factor-based holistic intervention programs to reduce its incidence.

**Systematic review registration:**

The protocol of this study has been registered in the database PROSPERO (registration ID: CRD42023433578).

## 1 Introduction

In 2020, breast cancer became the most common malignant tumor in the world, surpassing lung cancer and ranking first in female cancer (1). The development of multimodality diagnosis and treatment has greatly improved the long-term survival rate of patients with breast cancer and has resulted in an increasing number of breast cancer survivors. Currently, the 5- and 10-year survival rates of patients with breast cancer are 90% and 80%, respectively (2). Consequently, quality of life has become an important measure of patient outcomes in modern oncology (3). Mitchell et al. (4) showed that 30%–40% of patients with cancer had psychological problems, which led to a decrease in treatment compliance, medical satisfaction, and quality of life.

The International Psycho-Oncology Society (5) identified psychological distress (PD) as the sixth most important vital sign in 2010 and included PD assessment as a routine item in clinical care practice. The National Comprehensive Cancer Network (NCCN) (6) describes cancer-related distress as a complex and unpleasant combination of psychological (such as cognitive, behavioral, and emotional), social, and spiritual challenges that may impact an individual’s ability to effectively cope with the physical symptoms of cancer and its treatment. This suggests that PD should be rapidly identified, managed, recorded, and treated at any stage of cancer, especially when the condition changes. Furthermore, PD should be treated according to clinical guidelines. Unmanaged PD negatively affects cancer-related morbidity, mortality, and quality of life (7, 8). Marco and White (9) showed that patients with cancer have higher levels of PD than the normal population, with approximately 21% and 13% of patients suffering from anxiety and depression, respectively, causing a poorer quality of life.

In recent years, the literature on the incidence and risk factors of PD in patients with cancer has increased. In Denmark, 8% of women experienced severe distress throughout the first eight months following diagnosis (10). In Korea, 19.4% of patients with breast cancer were in a state of continuous high PD one year after diagnosis (11). The latest systematic review from 2020 found that the pooled prevalence of PD from 17 studies covering 3,870 patients with breast cancer was 50% (12). However, this systematic review does not distinguish between the sexes, yet male and female patients with breast cancer have different epidemiological patterns, risk factors, and diagnostic features. Further, their molecular and clinicopathological features, as well as personality traits and psychological characteristics are significantly different, and their PD may also be different (13, 14). Compared with male breast cancer, female breast cancer has a higher incidence rate (1). It is meaningful to carry out a systematic review based on female PD. In addition, the review mainly assesses English literature. Although this can reflect the incidence rate of PD in breast cancer patients to a large extent, considering that China’s breakthrough breast cancer population accounts for a considerable proportion of that of the world (1), the inclusion of Chinese literature could increase the persuasiveness of the results. Finally, although this systematic review confirms that the distress thermometer (DT) is a fast, self-reported distress screening tool for cancer patients, it does not it does not further explore the influencing factors of PD evaluated through this tool. Another systematic review from 2016 (15) explored the predictive factors of PD from the perspective of female patients with breast cancer; however, there is no consensus on the definition of PD in the included literature. Multiple measurement tools, such as the DT, Hamilton Anxiety and Depression Scale, Hospital Anxiety and Depression Scale, are used to measure PD, which may lead to heterogeneity in the final results.

The DT is a simple and convenient scale that has been widely used to screen for PD in patients with cancer. Its effectiveness and reliability have been tested in many countries and regions (16, 17). However, the optimal cutoff value of the scale remains disputed (18). In different studies, the optimal threshold for DT ranged from four to seven points (12). For breast cancer, studies in Denmark and the United States recommended a score of seven as the best cutoff to define PD, whereas studies in Indonesia recommended five as the best cutoff score (19–21). These differences are caused by variations in the cultural backgrounds, lifestyles, and expressions of different regions; for example, Western culture tends to be more extroverted, whereas Eastern culture is more introverted. Therefore, it is crucial to conduct a systematic review of studies on PD assessment among patients with breast cancer using DT diagnostic tools, which will help to clearly and more comprehensively understand the current situation of this population.

Some extant literature has reported many factors that increase the PD of patients with breast cancer. This includes demographic characteristics, such as age (22, 23) and education level (24); sociological characteristics, such as economic issues (25) and healthcare payment status (24); and disease status, such as oncological staging (23, 26), treatment type (27), and social support level (28). However, other studies have not found these associations (29, 30). Thus, elucidating the incidence and influencing factors of PD can help raise the awareness of PD among healthcare professionals and provide guidance and reference for developing targeted and optimal intervention measures. Through this study, we aimed to (1) determine the incidence of PD in women with breast cancer and (2) determine the predictive factors of PD.

## 2 Methods

### 2.1 Protocol and registration

This review was conducted in accordance with the Meta-Analysis of Observational Studies in Epidemiology guidelines. The detailed study protocol is available on the PROSPERO website under the registration number CRD42023433578.

### 2.2 Search strategy

We systematically searched the following databases for through April 2023: PubMed, Embase, Cochrane Library (CENTRAL), CINAL (via EBSCO), PsycINFO, China Knowledge Resource Integrated Database (CNKI), Wanfang Database, and the Chinese Biomedical Database (CBM), and Weipu Database (VIP). The search strategies were performed using a combination of mesh terms and free words. Search strings contained the terms “breast neoplasms,” “breast neoplas*,” “breast tumor*,” “breast cancer,” “breast carcinoma*,” “mammary cancer*,” “mammary carcin*,” “mammary neoplas*,” “breast metasta*,” “breast malig*,” “breast malignant neoplas*,” “malignant neoplasm of breast,” “breast malignant tumor*,” “malignant tumor of breast,” and “psychological distress,” “psychiatric distress,” “emotional distress,” emotional stress,” “mental distress,” “distress thermometer,” “distress symptom,” “distress;” and “risk factors,” “risk factor*,” “risk*,” “predictor,” “predictive factor,” “influence factor,” “correlat*,” “predict*,” “prevalence,” “incidence,” “incident,” “epidemiology,” “rate,” “frequency,” “occurrence,” “morbidity,” “proportion,” and “probability.” The precise search strategies for the English databases are presented in the Appendix (**Supplementary Table 1**). The reference lists included in the identified articles were manually searched for additional relevant publications.

### 2.3 Study selection

After removing duplicate studies, two investigators independently assessed eligible publications by screening titles and abstracts according to the inclusion and exclusion criteria. When at least one reviewer decided that an abstract was eligible for inclusion, full texts and articles were retrieved. Each publication was independently assessed by both investigators for inclusion in the final study. Disagreements were resolved through discussions.

The inclusion criteria were as follow: (1) cross-sectional studies and cohort studies; (2) study participants were adult (age ≥ 18 years) females diagnosed with breast cancer; (3) prevalence and/or risk factors of PD in patients with breast cancer; and (4) PD was evaluated using the DT, which was recommended by the NCCN Cancer Clinical Guidelines. The DT scoring system is similar to the classic visual distress scoring method, with 0 representing “no PD” and 10 representing “extreme PD.” The guidelines recommend considering PD of at least 4 points as clinically significant. However, they do not provide a clear definition of the scores for mild, moderate, severe, and extremely severe PD. Consequently, different countries have inconsistent classifications of DT. Most studies classify DT of at least 3 or 4 points as moderate distress, and at least 6 or 7 as severe. No language restrictions were applied to eligible studies. The exclusion criteria were as follows: (1) conference abstracts, reviews, and study protocols; (2) unavailable full text; (3) sample size below 50, which is deemed small by the British statistician Gosset (31). A study with a relatively small sample size is more likely to lack sufficient statistical power to detect the true positive association if the result is negative; when the result is positive, the finding could possibly be due to the smaller sample size; and (4) low research quality.

### 2.4 Data extraction

Two authors independently extracted the following data using a data extraction form developed *a* priori: (1) study characteristics, including author name, title, and year of publication; (2) population characteristics, including country, age, sample size, and the initial time of distress identified; and (3) DT cutoff score and prevalence of PD in breast cancer. The risk factors of PD, odds ratios (ORs), and 95% confidence intervals (CIs) of the independent risk factors for distress in breast cancer were extracted. If outcome data were unclear or not reported, we contacted the authors to obtain missing data. Disagreements were resolved through consensus or by a third author.

### 2.5 Quality appraisal

Two authors independently assessed the quality of the included studies, as previously described. The quality of cross-sectional studies was assessed using an 11-item checklist, which was recommended by the Agency for Healthcare Research and Quality (AHRQ) (32). Each item is scored as “1” if it was answered “YES” and “0” if it was answered “NO” or “UNCLEAR.” The highest score is 11, with the quality level being assessed as follows: low quality=0–3; moderate quality=4–7; and high quality=8–11. The AHRQ results showed that the included studies scored between 5 and 9.

The Newcastle–Ottawa Quality Scale (NOS) (33) was used to assess the quality of the cohort studies, including study participant selection, intergroup comparability, and exposure factors. The NOS includes three domains and eight items, of which 9 points are full. A score of 5 was considered high quality. Two reviewers independently evaluated the included studies. They discussed the results and arrived at a consensus on each item of the checklist for each study. The results of the NOS evaluation showed that all the included studies had a score of ≥5 and were of high quality. Four studies were rated full marks. The detailed scores are shown in **Supplementary Tables 2**, **3**.

### 2.6 Statistical analysis

Statistical analyses were conducted using Stata 14 software and RevMan 5.3. The pooled prevalence and 95% CIs for PD were calculated using Stata 14, and the pooled risk factors and 95% CIs for PD were calculated using RevMan 5.3. Due to inconsistent cutoff values for DT included in the study, when combining the incidence of PD, when there was only one cutoff value, the incidence was directly included. When there were two cutoff values, the incidence of lower cutoff values was included. Statistical heterogeneity in the pooled results was assessed using the chi-square test, Cochran’s Q-test, and the inconsistency *I* ^2^ test, with *I* ^2^ values of 25%, 50%, and 75% indicating low, moderate, and high heterogeneity, respectively. Pooled prevalence, risk factors, and 95% CIs for PD were calculated using a random-effects model when Cochrane’s Q statistic detected significant heterogeneity, and they were shown in the forest plot. Subgroup analysis was performed to assess the incidence of PD in different DT cutoff scores, treatment phase at initial distress assessment, and countries, and the results were combined using the inverse variance method for ORs and 95% CIs. Descriptive analyses were performed for data that could not be combined. Publication bias was identified using a funnel plot, and asymmetry was tested using Egger’s linear regression method (p<0.1, considered significant).

## 3 Results

### 3.1 General results of the included studies

**Figure 1:**
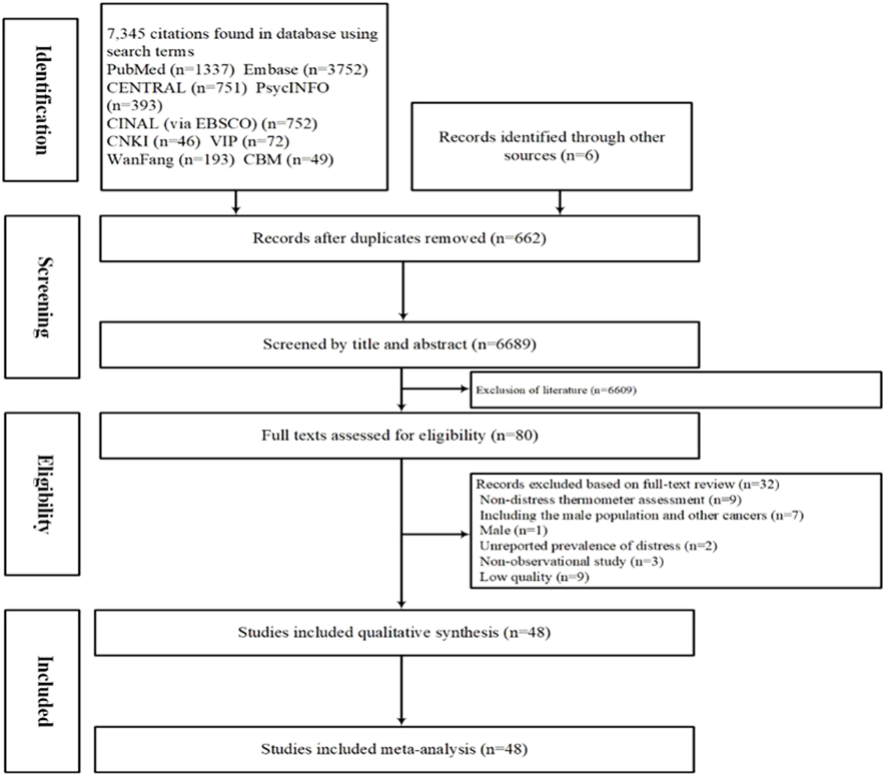
PRISMA flowchart of study selection.

The initial search retrieved 7,351 articles, of which 662 were duplicates. After excluding 6,689 studies based on their titles and abstracts, the full texts of 80 publications were examined. Of these, 48 (13 in Chinese and 35 in English) met the inclusion criteria and were therefore included in the meta-analysis (**Figure 1**).

### 3.2 Characteristics of the included studies

The characteristics of the 48 analyzed studies are summarized in **Table 1**. The literature was published between 2006 and 2023, the sample sizes ranged from 77 to 1,400, with mean ages ranging from 45.1 to 77.0 years. Forty-seven studies with 15,157 adult female breast cancer in total reported the prevalence of PD. Thirteen studies reported risk factors for PD. In addition, regarding geographic regions, 19 studies were from Asia, 18 from Europe, 10 from North America, and 1 from Africa. Concerning the treatment phase at initial distress assessment, the studies included newly diagnosed breast cancer patients, breast cancer survivors, and those receiving surgical treatment, adjuvant therapy.

**Table 1:** Characteristics of included studies.

Authors	Years	Study setting	Study design	Sample size	Mean age	Treatment phase at initial distress assessment	Prevalence (%)	DT cutoff	Risk of bias	Risk factors assessed
Hegel et al. (34)	2006	USA	Cross-sectional	236	57.4±12.3	Newly diagnosed	40.7	>5	6	-
Hegel et al. (35)	2008	USA	Cross-sectional	321	57.8±12.6	Newly diagnosed	25.3	≥7	8	-
Luutonen et al. (36)	2011	Finland	Cross-sectional	276	57.8±9.90	Adjuvant treatment	28.4	≥4	8	-
Bidstrup et al. (37)	2012	Denmark	Cross-sectional	333	60.0±10.0	Newly diagnosed	43.2	≥7	7	-
Mosher et al. (38)	2012	USA	Cross-sectional	173	NR	Mixed treatment phases	58.4	≥4	7	-
Mertz et al. (39)	2012	Denmark	Cross-sectional	343	60.0	Newly diagnosed	76.7 (DT≥3) 43.2 (DT≥7)	≥3 ≥7	6	-
Head et al. (40)	2012	USA	Cross-sectional	102	52.7±11.7	Newly diagnosed	64.7	≥4	8	-
Agarwal et al. (41)	2013	USA	Cross-sectional	229	56.0±11.0	Mixed treatment phases	57.6 (DT≥4) 16.2 (DT≥7)	≥4 ≥7	7	Emotional, physical, and spiritual concerns; depression, recent diagnosis, and unemployment
Ploos et al. (29)	2013	Netherlands	Cross-sectional	129	57.0±10.0	Mixed treatment phases	36.4	≥5	7	-
Schmid al (42).	2013	Switzerland	Cross-sectional	175	57.5±11.4	Mixed treatment phases	56.2	≥4	7	-
Mejdahl et al. (43)	2015	Denmark	Prospective	335	61.0	Mixed treatment phases	76.7 (DT≥3) 43.3 (DT≥7)	≥3 ≥7	6	-
McFarland et al. (44)	2016	USA	Cross-sectional	98	55.4±13.2	Mixed treatment phases	55.1 (DT≥4) 17.4 (DT≥7)	≥4 ≥7	6	
Ploos et al. (45)	2016	Netherlands	Cross-sectional	181	55.0	Newly diagnosed	34.3	≥7	9	-
Lo et al. (46)	2016	Netherlands	Prospective	746	58.0	Mixed treatment phases	40.9	≥5	9	Lacking muscle strength, low level of life satisfaction, more frequent cancer worries, neuroticism
Jørgense et al. (47)	2016	Denmark	Prospective	1024	60.0±10.8	Newly diagnosed	68.9 (DT≥4) 39.8 (DT≥7)	≥4 ≥7	9	-
Xue et al. (48)	2016	China	Cross-sectional	196	48.3±8.2	Adjuvant treatment	54.6 (DT≥4) 11.2 (DT≥7)	≥4 ≥7	5	-
Park et al. (22)	2017	Korea	Prospective	117	45.1	Adjuvant treatment	18.8	≥4	9	Age, depression, neuroticism, pain,
Berhili et al. (25)	2017	Morocco	Cross-sectional	446	50.0±8.0	Mixed treatment phases	46.6	≥3	6	financial difficulty, age, absent family support, chemotherapy, surgery, distant metastasis
Mertz et al. (49)	2017	Denmark	Prospective	474	61.0	Newly diagnosed	35.2	≥7	7	-
Ng et al. (50)	2017	Malaysia	Prospective	211	55.0±11.5	Newly diagnosed	50.2	≥4	7	-
Acquati et al. (51)	2017	USA	Retrospective	89	54.3±11.9	Mixed treatment phases	63.5	≥4	5	-
Robbeson et al. (52)	2018	Netherlands	Cross-sectional	90	59.8± 9.9	Survivorship	46.7	≥5	6	-
Li et al. (24)	2018	China	Cross-sectional	392	48.3±8.5	Newly diagnosed	62.2	≥4	6	Education level, no medical insurance, family relationship disharmony
Shen et al. (53)	2018	China	Prospective	240	50.3±10.5	Newly diagnosed	36.8	≥4	7	-
Zhang et al. (54)	2018	China	Prospective	113	52.3±6.1	Newly diagnosed	43.4	≥4	6	-
Cormio et al. (55)	2019	Italy	Cross-sectional	143	NR	Mixed treatment phases	48.3	≥6	6	-
Ciambella et al. (56)	2019	USA	Retrospective	474	63.0	Newly diagnosed	66.5	≥4	6	-
Wang et al. (57)	2019	China	Cross-sectional	245	49.3±12.2	Newly diagnosed	33.5	≥4	7	-
Wan et al. (58)	2019	China	Cross-sectional	210	49.5±8.4	Adjuvant treatment	73.3 (DT≥4) 24.7 (DT≥7)	≥4 ≥7	6	-
Yang et al. (23)	2019	China	Cross-sectional	193	53.3±11.3	Newly diagnosed	74.1	≥4	7	Age, breast conserving surgery, late stage
Civilotti et al. (59)	2020	Italy	Cross-sectional	436	57.2±12.5	Newly diagnosed	71.1	≥4	6	-
Fayanju et al. (60)	2020	USA	Retrospective	1029	58.0	Newly diagnosed	53.3	≥4	7	Emotional stressors, black patients
de Boer et al. (61)	2020	Netherlands	Prospective	85	77.0	Mixed treatment phases	64.0	≥4	7	-
Admiraal et al. (62)	2020	Netherlands	Prospective	77	52.1	Adjuvant treatment	45.5	≥5	7	-
Li et al. (63)	2020	China	Prospective	117	45.8±8.7	Mixed treatment phases	62.4	≥4	9	Choleric temperament, sanguine temperament, phlegmatic temperament, part-time job, retire
Sun et al. (26)	2020	China	Cross-sectional	172	49.3±10.3	Mixed treatment phases	49.4	≥4	8	Age, late stage, short disease course, low monthly household income
Liu et al. (64)	2021	USA	Retrospective	773	54.0	Mixed treatment phases	21.3	≥5	5	-
Budisavljevic et al. (65)	2021	Croatia	Cross-sectional,	201	53.1	Mixed treatment phases	54.2	≥4	8	-
Wang et al. (66)	2021	China	Prospective	270	NR	Mixed treatment phases	62.2	≥4	7	Age, education level, low monthly household income, late stage, CFQ score, AAQ-II score, MAAS score
Zhao et al. (67)	2022	China	Cross-sectional	137	NR	Adjuvant treatment	42.3	≥4	8	-
Tu et al. (27)	2022	China	Retrospective	96	48.6±9.9	Mixed treatment phases	NR	≥4	5	Age, monthly income, no medical insurance, modified radical mastectomy, SAS score<50
Taurisano et al. (68)	2022	Italy	Retrospective	150	59.4±13.2	Mixed treatment phases	86.7 (DT≥4) 40.7 (D>7)	≥4 >7	6	-
Lim et al. (69)	2022	Singapore	Retrospective	1238	NR	NR	40.0	≥4	7	-
Hass et al. (70)	2022	Germany	Prospective	1400	51.8±9.7	Mixed treatment phases	62.3 (DT≥5) 41.9 (DT≥7)	≥5 ≥7	5	-
Lv et al. (71)	2022	China	Prospective	96	NR	Adjuvant treatment	49.0	≥4	7	-
Liu et al. (72)	2022	China	Prospective	166	48.7± 9.4	Mixed treatment phases	57.8	≥5	7	Emotional concerns, practical problems, yield
Wang et al. (73)	2022	China	Cross-sectional	258	48.5±8.0	Mixed treatment phases	67.4	≥4	7	Age, ethnic minorities, monthly household income, economic burden caused by disease, modified radical mastectomy
Pang et al. (74)	2023	China	Cross-sectional	122	NR	Survivorship	50.8	≥4	7	-

### 3.3 Prevalence of psychological distress

**Figure 2:**
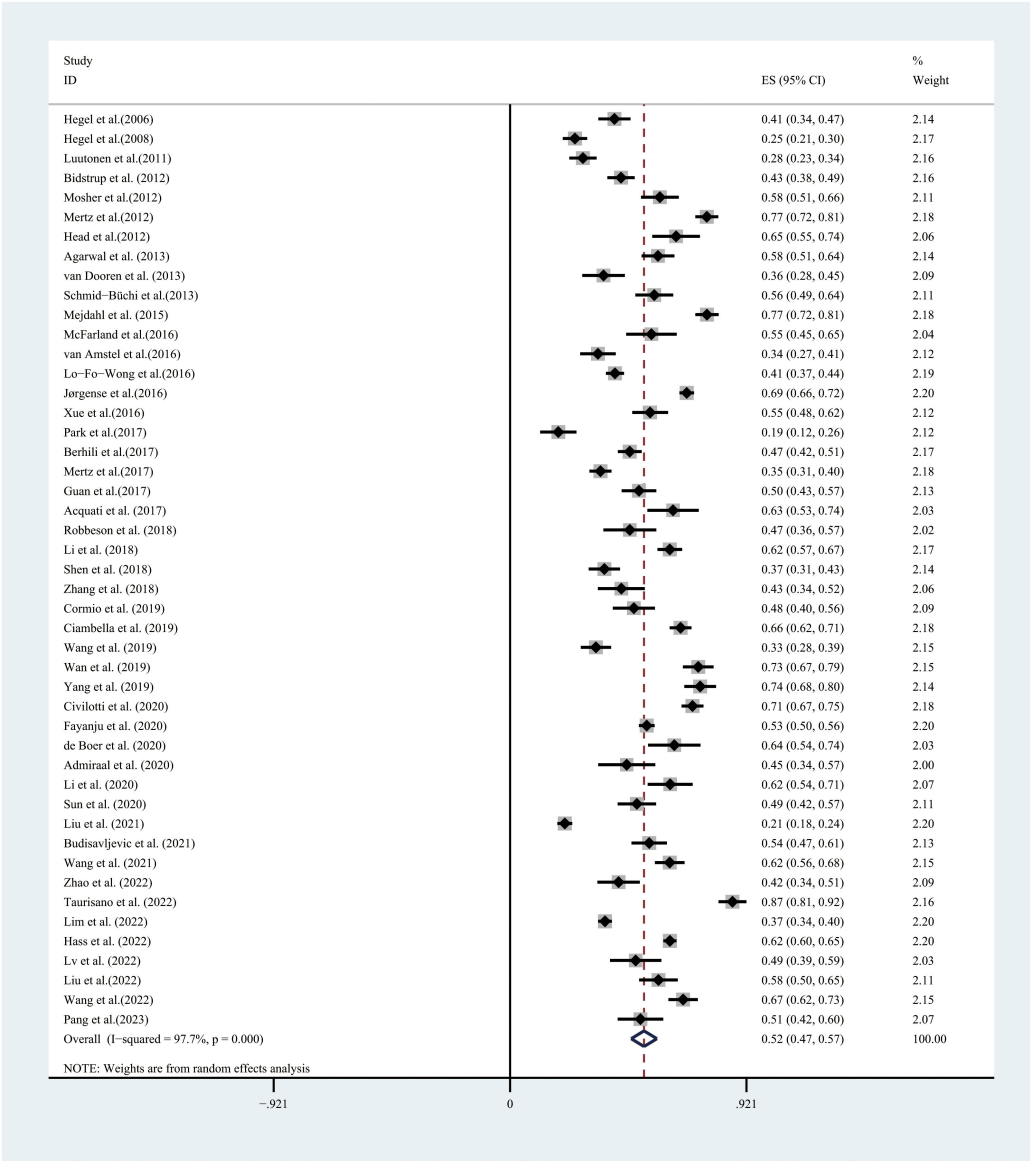
Forest plot of prevalence of psychological distress.

In the 47 studies available for the meta-analysis, the prevalence of PD, assessed using the DT, ranged between 11.2%–86.7%. PD, based on a random-effects model, showed that the overall PD prevalence was 52.0% (95% CI:47.0%–57.0%, *I* ^2^ = 97.7%, *P*=0.000) (**Figure 2**).

### 3.4 Subgroup prevalence of psychological distress

A subgroup analysis was conducted to explore the heterogeneity between studies. Those with different DT cutoff scores (DT≥3, DT≥4, DT≥5, DT≥6, and DT≥7), countries (American, Asian, European, and African), and treatment phase at initial distress assessment (newly diagnosed, adjuvant treatment, mixed treatment phases, and survivorship) were grouped and analyzed separately; the pooled estimates showed that the pooled prevalence of PD was similar within subgroups. In the random-effects model, the estimates of pooled prevalence were calculated for different DT cutoff scores; DT≥3, DT≥4, DT≥5, DT≥6, and DT≥7 were 67.0%, 55.0%, 44.0%, 44.0%, and 32.0%, with high heterogeneity observed between studies (*I* ^2^ = 98.2%, *I* ^2^ = 96.7%, *I* ^2^ = 98.7%, *I* ^2^ = 51.7%, *I* ^2^ = 95.8%). The estimated pooled prevalence rates of PD in American, Asian, European, and African countries were 50.0%, 51.0%, 54.0%, and 46.0%, respectively, with high heterogeneity observed between studies. The estimated pooled prevalence rates of PD were 53.0% for newly diagnosed participants, 45.0% for those undergoing adjuvant treatment, 55.0% for mixed treatment phase patients, and 49.0% for survivorship, but with significant heterogeneity. The estimated pooled results obtained from the subgroup analyses are shown in **Table 2**. A forest plot of the subgroup analysis results is presented in the **Supplementary Materials (Supplementary Figures 1**–**3)**.

**Table 2:** Subgroup analyses based on different DT cutoff score, countries, and stage of breast cancer.

Subgroups	Number of included studies	Sample size	Psychological distress				
			Prevalence	95% CI	*I* ^2^		*P* value
DT cutoff score							
DT≥3	3	1124	67.0%	47.0%-86.0%	98.2%		0.000
DT≥4	31	8868	55.0%	50.0%-61.0%	96.7%		0.000
DT≥5	7	2366	44.0%	29.0%-59.0%	98.7%		0.000
DT≥6	2	393	44.0%	37.0%-51.0%	51.7%		0.150
DT≥7	13	4197	32.0%	26.0%-38.0%	95.8%		0.000
Countries							
America	10	3524	50.0%	38.0%-63.0%	98.2%		0.000
Asia	18	4493	51.0%	44.0%-59.0%	96.0%		0.000
Europe	18	6598	54.0%	46.0%-62.0%	97.8%		0.000
Africa	1	446	47.0%	–	–		–
Treatment phase at initial distress assessment							
Newly diagnosed	17	6347	53.0%	45.0%-61.0%	97.6%		0.000
Adjuvant treatment	7	1109	45.0%	29.0%-60.0%	96.7%		0.000
Survivorship	2	212	49.0%	42.0%-56.0%	0.0%		0.550
Mixed treatment phases	21	7393	55.0%	48.0%-63.0%	98.0%		0.000

### 3.5 Risk factors

The pooled analysis revealed that higher education level, late stage of the tumor, emotional concerns, no medical insurance, modified radical mastectomy, and history of depression were independent risk factors. Age and neuroticism were not associated with PD, and higher monthly income was a protective factor against PD. Descriptive analyses were used for data that could not be combined. Detailed results of the risk factors are presented in **Table 3**.

**Table 3:** Results of risk factors.

	Risk factor	Included studies	Sample size	OR (95% CI)
Patient characteristics	Age≥50	49 (11, 23, 27, 66)	676	OR=0.59 (0.32-1.09)
	Age<50	3 (25, 26, 73)	876	OR=0.82 (0.43-1.59)
	Higher education level	2 (24, 66)	662	OR=2.42 (1.83-3.18)
	Unemployed	1	229	OR=4.27 (1.22-14.88)
	Part-time job	1	117	OR=17.48 (1.19-257.72)
	Retire	1	117	OR=13.90 (1.14-169.14)
	Black patients	1	1029	OR=0.59 (0.41-0.83)
	Late stage of tumor	3 (23, 26, 66)	635	OR=4.39 (2.39-8.05)
	Short disease course	1	172	OR=0.37 (0.25-0.56)
	Ethnic minorities	1	258	OR=0.33 (0.11-0.93)
Patient concerns	Emotional concerns	3 (41, 60, 72)	1424	OR=3.24 (2.00-5.27)
	Physical concerns	1	229	OR=1.82 (1.35-2.44)
	Spiritual concerns	1	229	OR=5.76 (1.39-23.94)
	Practical problems	1	166	OR=1.59 (1.15-2.20)
	More frequent cancer worries	1	746	OR =1.40 (1.05-1.89)
Economic situation and Family	Financial difficulties	1	446	OR=1.95 (1.03-3.70)
	Low monthly income	1	270	OR=2.02 (1.53-2.51)
	Economic burden caused by disease	1	258	OR=6.10 (1.68-26.52)
	Higher monthly income	3 (26, 27, 73)	526	OR=0.19 (0.06-0.59)
	NO Medical insurance	2 (24, 27)	488	OR=3.31 (2.43-4.52)
	Absent Family support	1	446	OR=6.82 (3.31-15.32)
	Family relationship disharmony	1	392	OR=3.33 (2.48-5.97)
Treatment	Breast conserving surgery	1	193	OR=0.17 (0.05-0.55)
	Modified radical mastectomy	3 (23, 27, 73)	547	OR=2.74 (1.59-4.71)
	Chemotherapy	1	446	OR=3.16 (1.50-6.70)
	Distant metastasis	1	446	OR=7.04 (2.50-19.85)
Assessment	Higher CFQ score	1	270	OR=3.55 (2.75-4.34)
	Higher AAQ-IIscore	1	270	OR=2.07 (1.24-2.90)
	Higher MAAS score	1	270	OR=0.85 (0.73-0.97)
	SAS score<50	1	96	OR=0.18 (0.08-0.69)
Others	History of depression	2 (11, 41)	346	OR=4.06 (1.84-8.97)
	Neuroticism	2 (11, 46)	863	OR=2.54 (0.31-21.11)
	Pain	1	117	OR=6.74 (1.10-41.05)
	Lacking muscle strength	1	746	OR=1.82 (1.12-2.98)
	Diagnosed 31–350 days	1	229	OR=0.35 (0.17-0.73)
	Low level of life satisfaction	1	746	OR=0.77 (0.67-0.89)
	Choleric temperament	1	117	OR=0.02 (0.01-0.62)
	Sanguine temperament	1	117	OR=0.01 (0.00-0.07)
	Phlegmatic temperament	1	117	OR=0.05 (0.00-0.52)
	Coping style (yield)	1	166	OR=1.30 (1.14-1.48)

### 3.6 Risk of bias in the included studies

**Figure 3:**
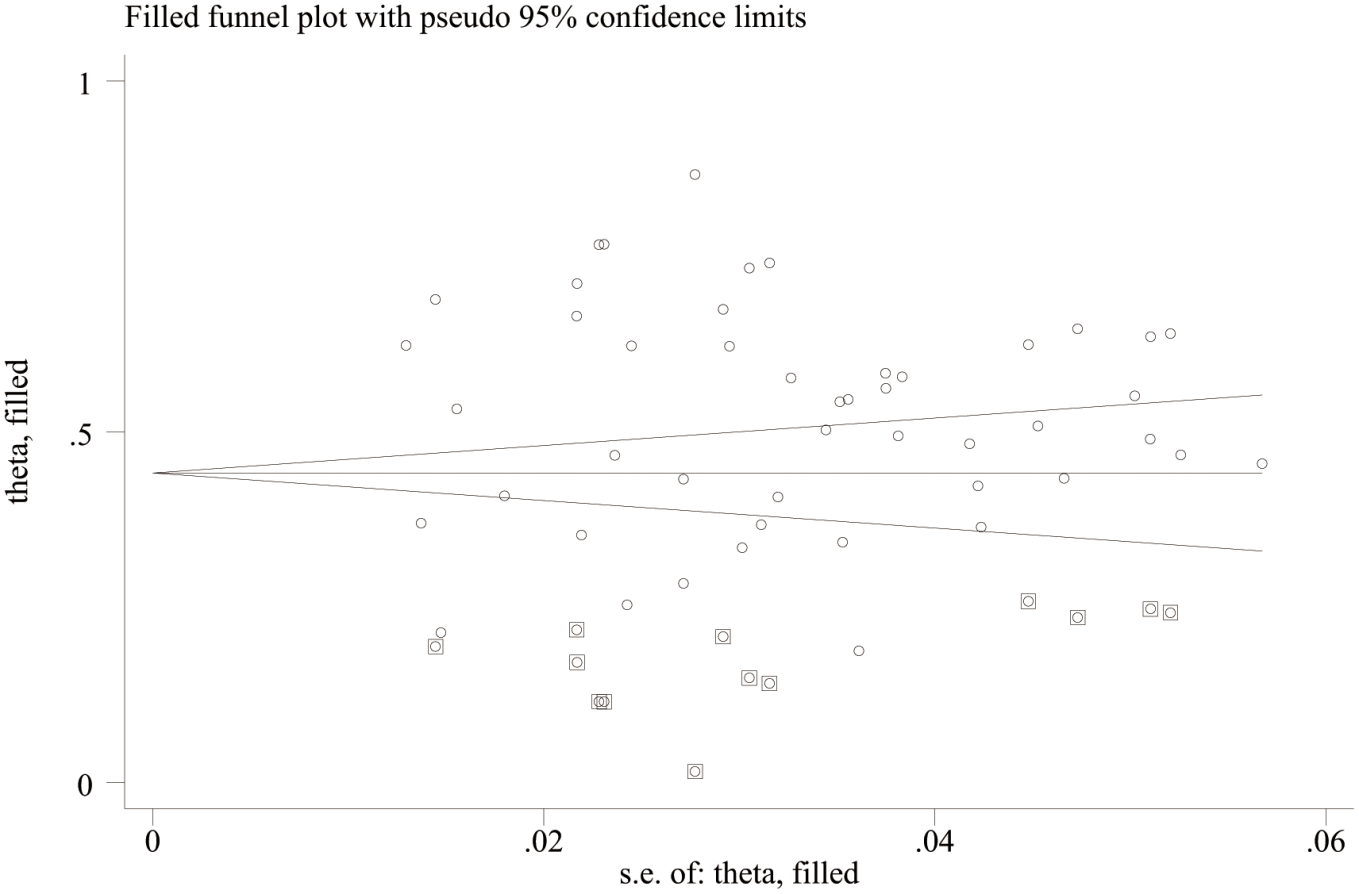
Funnel plot for the publication bias of included studies.

Publication bias analysis showed that there was no significant publication bias in the literature included in this study (Egger’s test, p = 0.763; Begg’s test, p = 0.673, **Figure 3**), indicating that, when compared with a single study, this study can more reliably reflect the PD of female patients with breast cancer and more objectively identify risk factors. There was no evidence of publication bias in the prevalence of PD.

## 4 Discussion

### 4.1 Resource identification initiative

This was a systematic review and meta-analysis of the incidence and risk factors of PD in patients with breast cancer. The DT revealed a prevalence of PD ranging between 11.2%–86.7%, while the meta-analysis revealed an overall estimated 52.0% prevalence of PD among female patients with breast cancer. Further, this study analyzed 10 potential risk factors and revealed that higher education level, late-stage tumor, emotional concerns, no medical insurance, modified radical mastectomy, and history of depression were independent risk factors. Age and neuroticism were not associated with PD, and higher monthly income was a protective factor for PD.

### 4.2 Prevalence of psychological distress

This study shows that female patients with breast cancer have a high prevalence rate of PD, and that the prevalence rate of PD varies across different regions. This indicates that the detection rate of PD in female patients with breast cancer may differ owing to the selection of samples, cultural differences, economic and social conditions, and other factors. The pooled prevalence rates of PD among newly diagnosed patients, patients undergoing adjuvant treatment, and survivorship were 53.0%, 45.0%, and 49.0%, respectively. A higher degree of PD in newly diagnosed patients was consistent with the findings of Ribnikar et al. (75). Most patients may be in denial at the time of diagnosis, which increases their negative emotions; the patients do not know much about cancer, malignancy, and aggressiveness, and the uncertainty of the disease is the main reason for PD (76, 77). However, the adjuvant phase is a period of high concern for current medical workers, and a range of care measures have been developed to alleviate the PD of patients with breast cancer who are in the radiotherapy and chemotherapy phases (78, 79). Relatively few interventions have been developed for the PD of newly diagnosed patients. Owing to limited medical resources, newly diagnosed patients often need to wait for admission to hospitals to receive treatment (80). Outside the hospital, it is difficult for nurses to intervene and mobilize family and community resources to manage the PD of newly diagnosed patients with breast cancer; thus, this should become the focus of future research.

### 4.3 Risk factors of psychological distress

#### 4.3.1 Demographic variables

Age is not associated with PD. As such, PD management interventions targeting different age groups may not significantly reduce the PD of female patients with breast cancer. A higher education level is identified to be an independent risk factor for PD. People with higher education levels possess a better understanding of the occurrence, development, prognosis, and potential harm of diseases in various ways and are especially sensitive to disease prognoses, leading to heavier psychological burden (81). Medical insurance was not identified as a risk factor for PD. The treatment cycle of breast cancer is long, and as treatment becomes more extensive, the associated costs also increase, causing financial difficulties for patients (82). Patients with poor economic conditions may fear missing the optimal time for diagnosis and treatment, leading to heightened negative psychological emotions (83). By contrast, patients with good economic status can access more medical and social resources, resulting in lower levels of PD (84). Lack of medical insurance places greater economic pressure on patients and increases their psychological and spiritual burdens, further exacerbating their PD (85, 86). Higher monthly income was found to be a protective factor against PD. Furthermore, Tao et al. (87) revealed that 43.9% of patients with breast cancer were unemployed after diagnosis. Therefore, implementing various measures to promote the return of such patients to work and increase family income is crucial (88).

#### 4.3.2 Clinical variables

Patients with late-stage tumors were found to be more prone to PD. Iwatani et al. (89) reported that tumor staging was an important predictor of PD, and patients with late-stage breast cancer were more likely to experience PD than those with early-stage cancer, consistent with the present study’s findings. There is a consensus that later stages have a worse prognosis (90). Terminal patients often experience poor treatment effects, leading to profound PD (91). Doctors and nurses can collaborate with psychotherapists and social groups for strengthening psychological intervention to address these negative experiences of PD. A history of depression is an independent risk factor for PD. Patients with a history of depression experienced more severe PD than patients with cancer. Another risk factor is modified radical mastectomy. In modified radical mastectomy, a procedure for breast cancer patients, the mammary glands, including the nipple areola complex, are removed (92). Moreover, during the procedure, there are different levels of lymph node dissection according to the particular stage of the disease (93). The surgical trauma is large, and adverse events related to wound healing are likely to occur after the surgery (94, 95). This may cause patients undergoing modified radical surgery to experience a higher level of physical pain. Additionally, post-operative visual defects of the breast may inevitably cause significant psychological trauma (96, 97). These outcomes may be important factors related to psychological distress in patients undergoing modified radical surgery for adenocarcinoma. Thus, healthcare professionals should provide psychological counseling and psychological support to patients undergoing modified radical surgery as early as possible.

#### 4.3.3 Psychosocial variables

Neuroticism was not found to be associated with PD. This conclusion was drawn mainly after merging the results of two articles (i.e., 11, 46). Due to limitations in the quality and quantity of studies included, this conclusion still requires more high-quality literature to support it. Theoretically, neuroticism is a significant predictor of adverse psychological outcomes in patients with cancer (98). People with neurotic personalities experience intense negative emotions in the face of difficulties such as a breast cancer diagnosis and its treatment—a state that may contribute to PD (99, 100). Emotional concerns have been identified as risk factors for PD. Breast cancer patients will suffer the first major blow when diagnosed; after diagnosis, they usually receive comprehensive treatment such as surgery, radiotherapy and chemotherapy (11). The pain of treatment, changes in physical appearance, and a series of other adverse reactions (e.g., related to high costs, aesthetic problems, cognitive impairment, sexual dysfunction) lead to the continuous negative emotions of most breast cancer patients after diagnosis (91). Therefore, it is crucial to provide continuous emotional support to manage the PD of breast cancer patients. On the one hand, clinical medical staff should timely assess breast cancer patients’ emotional problems, dynamically screen their psychological pain risk, and promptly refer them to professional psychologists or psychological consultants when necessary (101); On the other hand, various psychological intervention measures can be adopted, such as cognitive training (102), mindfulness meditation training (103), music therapy (79), etc., to improve individual perceptual sensitivity, enhance emotional regulation ability, enhance focus, and accept oneself.

### 4.4 Implications

Considering the high prevalence rate of PD (52.0%) uncovered in this study, it is necessary to raise clinicians’ awareness of the PD of women with breast cancer and arouse their attention to the urgency with which it should be addressed. Further, this study uncovered the predictive factors of PD of women with breast cancer. Specifically, higher education level, late-stage tumor, emotional concerns, no medical insurance, modified medical were identified as risk factors of PD; higher monthly income was identified as a protective factor against PD. Therefore, in the future, management of these factors, especially those that are controllable, such as emotional concerns, should be integrated into efforts to manage the PD of women with breast cancer. Clinicians are further recommended to incorporate initial screening and daily dynamic assessment of PD into clinical pathway management. Additionally, measures such as cognitive training (91), mindfulness meditation training (101), and music therapy (78), which are increasingly being used to treat emotional concerns in cancer patients and have been proven effective, should be used to supplement their treatment. Moreover, as a protective factor, higher monthly income may be difficult to control, but clinicians could provide social fund support channels for patients.

### 4.5 Limitations

This study had some limitations. First, some of the 48 included studies were of average quality, as the availability of high-quality studies was limited. Second, some included studies were a cross-sectional design; therefore, we do not know precisely about the direction of the association for some of the risk factor. Third, although sensitivity analyses and subcomponents were performed in this study, a considerable amount of heterogeneity was present among the studies, and some studies’ underlying characteristics were unclear, leading to analysis limitations. Especially, although this study has confirmed that the incidence of PD of breast cancer patients differs across countries, considering that there are too few studies in individual countries, such as Africa, it did not further analyze the incidence of PD of breast cancer patients in different countries under different cutoff values. Future research can explore this area to strengthen the results. Forth, the population of men with breast cancer is significantly smaller than that of women with breast cancer; hence, this study excluded men with breast cancer, potentially missing important information. For example, men with breast cancer may experience higher levels of PD and may thus require more attention. Finally, this research included studies using only the DT as the PD assessment tool. Therefore, future research should explore more psychological problems identified using other evaluation tools.

## 5 Conclusions

The current analysis indicates an overall pooled prevalence of PD of 52.0%, highlighting the necessity of evaluating and managing the PD of female patients with breast cancer. This systematic review has also established a set of evidence-based predictors that can be used to identify females at higher risk of experiencing PD. For example, higher education level, late-stage tumor, emotional concerns, no medical insurance, and modified radical were identified as independent risk factors. Higher monthly income was revealed as a protective factor against PD, suggesting that it is meaningful to directly provide financial support to patients with breast cancer or encourage them to return to work. Understanding the risk and protective factors of PD can help healthcare personnel manage the PD and treatment of female patients with breast cancer. Furthermore, these results can provide useful information for the development of a risk stratification algorithm for female breast cancer patients’ PD. This algorithm could help identify women with a high risk of suffering PD, thus aiding in the accurate prediction and early intervention of PD.

## Author contributions

LT: Writing – review & editing, Writing – original draft, Software, Methodology, Formal analysis, Data curation, Conceptualization. YX: Writing – review & editing, Writing – original draft, Software, Methodology, Formal analysis, Data curation, Conceptualization. XZ: Writing – original draft, Methodology. LF: Writing – original draft, Validation, Methodology. JL: Writing – original draft, Validation, Methodology. HC: Writing – original draft, Supervision, Conceptualization.
