# Supplementary figures and images for: Incidence and risk factors for psychological distress in adult female patients with breast cancer: a systematic review and meta-analysis
Source: Front Psychiatry. 2024 Mar 13;15:1309702. doi: 10.3389/fpsyt.2024.1309702 (PMC10965559; doi:10.3389/fpsyt.2024.1309702)

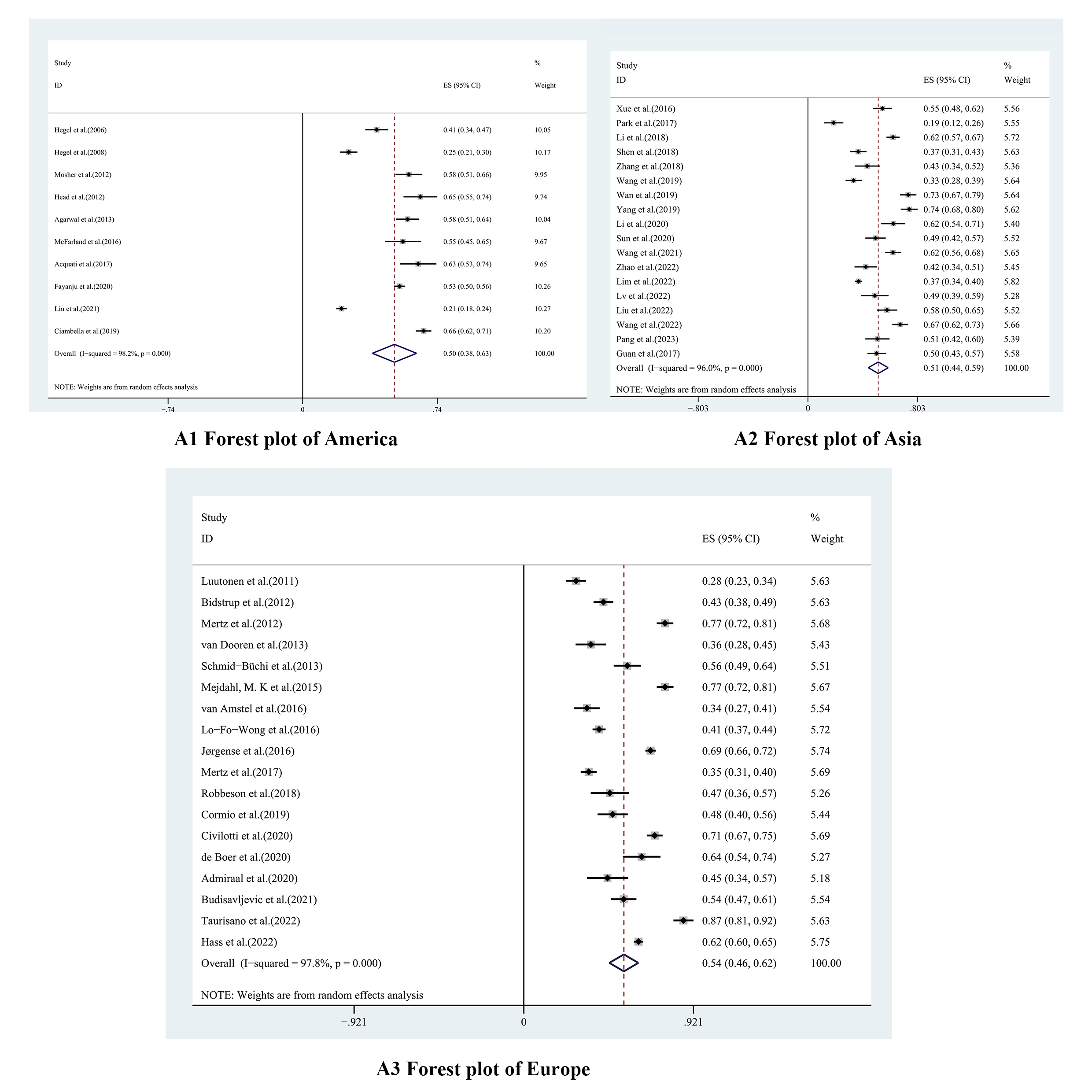

Supplement: Supplementary file 1 [file Image_1.tif]

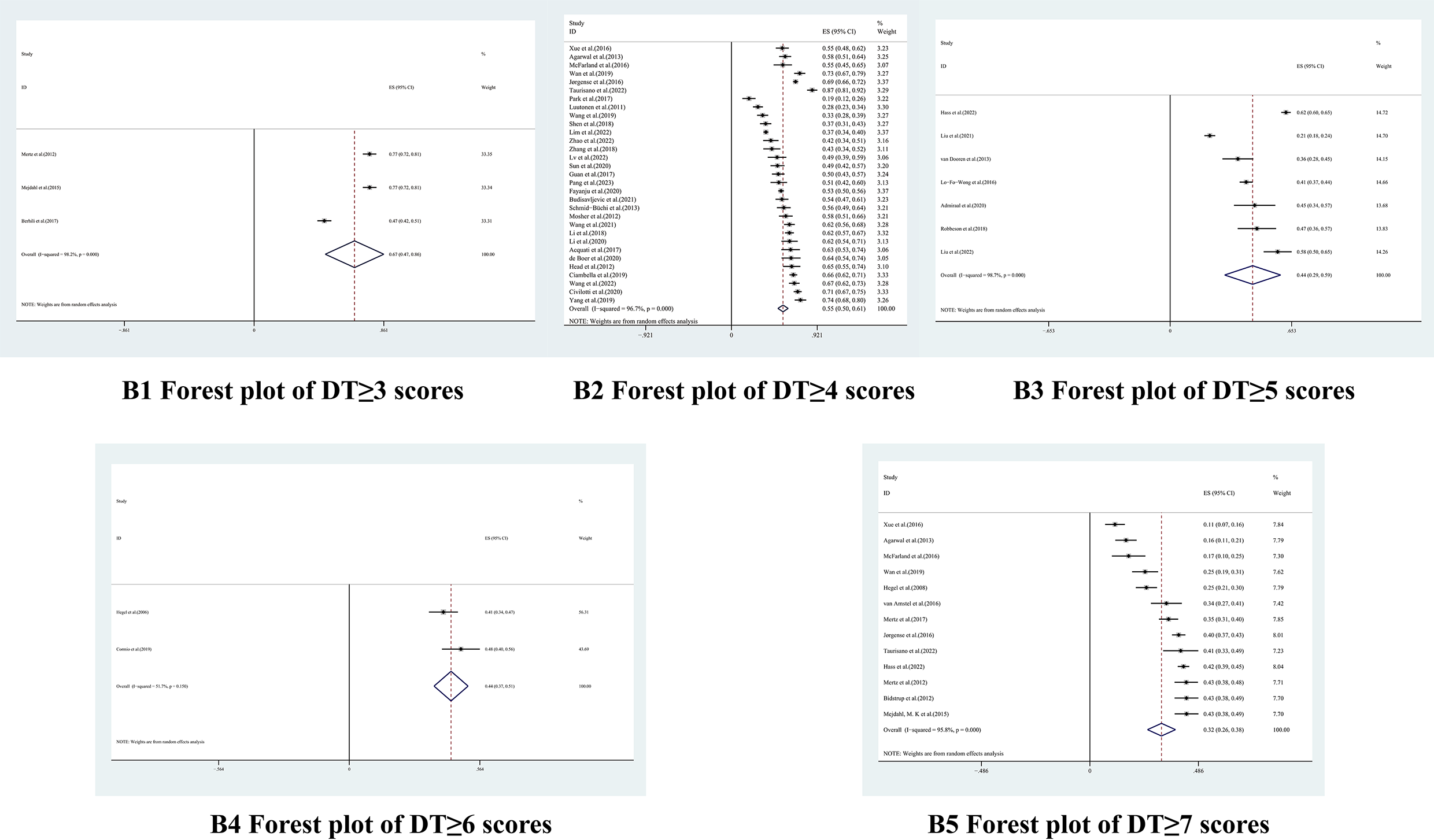

Supplement: Supplementary file 2 [file Image_2.tif]

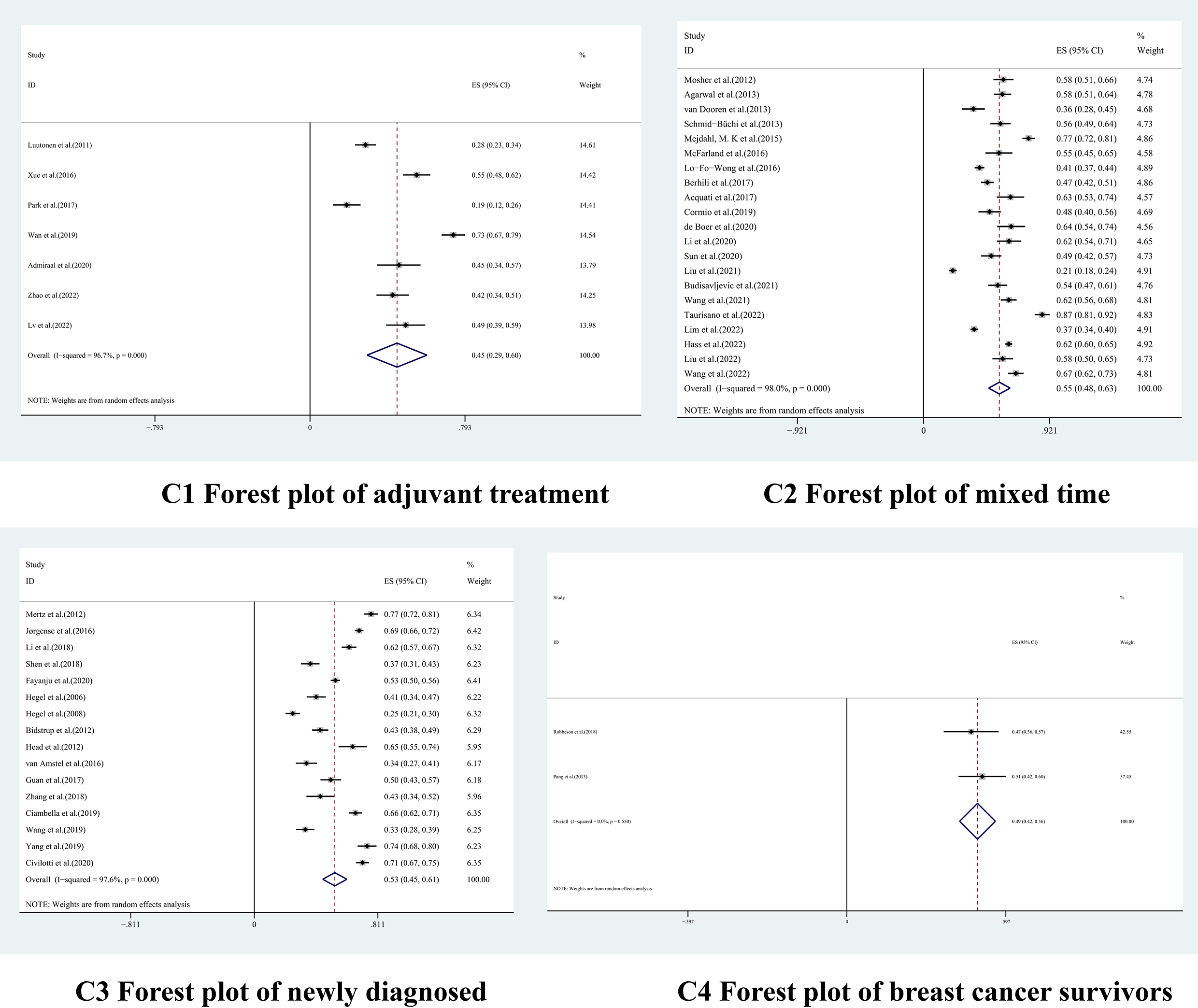

Supplement: Supplementary file 3 [file Image_3.tif]
