# Supplementary material for: Incidence and risk factors for psychological distress in adult female patients with breast cancer: a systematic review and meta-analysis
Source: Front Psychiatry. 2024 Mar 13;15:1309702. doi: 10.3389/fpsyt.2024.1309702 (PMC10965559; doi:10.3389/fpsyt.2024.1309702)
Supplement: Supplementary file 4 [file Table_1.docx]

**Supplementary table 1 Search strategies**

1. **Pubmed（1337）**

#1 "Breast Neoplasms"[Mesh]（338905）

#2 ((((((((breast Neoplas*[Title/Abstract]) OR (breast tumor*[Title/Abstract])) OR (breast cancer[Title/Abstract])) OR (breast Carcinoma*[Title/Abstract])) OR (mammary cancer*[Title/Abstract])) OR (mammary carcin*[Title/Abstract])) OR (mammary neoplas*[Title/Abstract])) OR (breast metasta*[Title/Abstract])) OR (breast malig*[Title/Abstract])（370965）

#3 (((Breast Malignant Neoplas*[Title/Abstract]) OR (Malignant Neoplasm of Breast[Title/Abstract])) OR (Breast Malignant Tumor*[Title/Abstract])) OR (Malignant Tumor of Breast[Title/Abstract])（65）

#4 #1 OR #2 OR #3（450837）

#5 "Psychological Distress"[Mesh] （6607）

#6  (((((((psychiatric distress[Title/Abstract]) OR (Emotional Distress[Title/Abstract])) OR (Emotional Stress[Title/Abstract])) OR (mental distress[Title/Abstract])) ) OR (distress thermometer[Title/Abstract])) OR (distress symptom[Title/Abstract])) OR (distress[Title/Abstract])（155,425）

#7 #5 OR #6（158,487）

#8 "Risk Factors"[Mesh]（952670）

#9 ((((((risk factor*[Title/Abstract]) OR (Risk*[Title/Abstract])) OR (Predictor[Title/Abstract])) OR (predictive factor[Title/Abstract])) OR (influence factor[Title/Abstract])) OR (correlat*[Title/Abstract])) OR (predict*[Title/Abstract])（2011136）

#10 (((((((((prevalence[Title/Abstract]) OR (incidence[Title/Abstract])) OR (incident[Title/Abstract])) OR (epidemiology[Title/Abstract])) OR (rate[Title/Abstract])) OR (frequency[Title/Abstract])) OR (occurrence[Title/Abstract])) OR (morbidity[Title/Abstract])) OR (proportion[Title/Abstract])) OR (probability[Title/Abstract])（5757496）

#11 #8 OR #9 OR #10（7585280）

#12 #4 AND #7 AND #11（1337）

1. **PsycINFO (393）**

S1 AB Breast Neoplasms OR AB breast Neopla* OR AB breast tumor* OR AB breast cancer OR AB breast Carcinoma* OR AB mammary cancer* OR AB mammary carcin* OR AB mammary neoplas* OR AB breast metasta* OR AB breast metasta* OR AB breast metasta* OR AB Malignant Neoplasm of Breast （21378）

S2 AB psychological distress OR AB psychiatric distress OR AB Emotional Distress OR AB Emotional Stress OR AB mental distress OR AB distress thermometer OR AB distress symptom OR AB mammary neoplas* OR AB distress （105968）

S3 AB Risk Factors OR AB risk factor* OR AB Risk* OR AB Predictor OR AB predictive factor OR AB influence factor OR AB correlat* OR AB predict* (1483819)

S4 AB prevalence OR AB incidence OR AB incident OR AB epidemiology OR AB rate OR AB frequency OR AB occurrence OR AB morbidity OR AB proportion OR AB probability (1030339)

S5 S3 OR S4 (2147018)

S6 S1 AND S2 AND S5 （393）

1. **CINAL（752）**

S1 AB Breast Neoplasms OR AB breast Neoplas* OR AB breast tumor* OR AB breast cancer OR AB breast Carcinoma* OR AB mammary cancer* OR AB mammary carcin* OR AB mammary neoplas* OR AB breast metasta* OR AB breast malig* OR AB Breast Malignant Neoplas* OR AB Malignant Neoplasm of Breast （53292）

S2 AB Breast Malignant Tumor* OR AB Malignant Tumor of Breast （437）

S3 S1 OR S2（53292）

S4 AB psychological distress OR AB psychiatric distress OR AB Emotional Distress OR AB Emotional Stress OR AB mental distress OR AB distress thermometer OR AB distress symptom OR AB distress （42910）

S5 AB Risk Factors OR AB risk factor* OR AB Risk* OR AB Predictor OR AB predictive factor OR AB influence factor OR AB correlate* OR AB predict* （870410）

S6 AB prevalence OR AB incidence OR AB incident OR AB epidemiology OR AB rate OR AB frequency OR AB occurrence OR AB morbidity OR AB proportion OR AB probability （877447）

S7 S5 OR S6（1390949）

S8 S3 AND S4 AND S7（752）

1. **CENTRAL（751）**

#1 MeSH descriptor: [Breast Neoplasms] explode all trees （17635）

#2 (breast cancer):ti,ab,kw OR (breast neoplas*):ti,ab,kw OR (breast carcin*):ti,ab,kw OR (breast Carcinoma*):ti,ab,kw OR (breast tumor*):ti,ab,kw (Word variations have been searched) （28556）

#3 ((breast* NEAR/5 neoplas*)):ti,ab,kw OR ((breast* NEAR/5 cancer*)):ti,ab,kw OR ((breast* NEAR/5 carcin*)):ti,ab,kw OR ((breast* NEAR/5 tumo*)):ti,ab,kw OR ((breast* NEAR/5 metasta*)):ti,ab,kw (Word variations have been searched)（43901）

#4 ((breast* NEAR/5 malig*)):ti,ab,kw OR (mammary cancer*):ti,ab,kw OR (Mammary Carcin*):ti,ab,kw OR (Mammary Neoplas*):ti,ab,kw (Word variations have been searched) （1947）

#5 #1 OR #2 OR #3 OR #4 （44527）

#6 MeSH descriptor: [Psychological Distress] explode all trees （402）

#7 (psychiatric distress):ti,ab,kw OR (Emotional Distress):ti,ab,kw OR (Emotional Stress):ti,ab,kw OR (mental distress):ti,ab,kw OR (distress thermometer):ti,ab,kw (Word variations have been searched) （14838）

#8 (distress symptom):ti,ab,kw OR (distress):ti,ab,kw (Word variations have been searched)（28471）

#9 #6 OR #7 OR #8 （34840）

#10 (Risk Factors):ti,ab,kw OR (risk factor*):ti,ab,kw OR (Risk*):ti,ab,kw OR (Predictor):ti,ab,kw OR (predictive factor):ti,ab,kw (Word variations have been searched)（302313）

#11 (influence factor):ti,ab,kw OR (correlat*):ti,ab,kw OR (predict*):ti,ab,kw (Word variations have been searched)（200519）

#12 (prevalence):ti,ab,kw OR (incidence):ti,ab,kw OR (incident):ti,ab,kw OR (epidemiology):ti,ab,kw OR (rate):ti,ab,kw（512202）

#13 (frequency):ti,ab,kw OR (occurrence):ti,ab,kw OR (morbidity):ti,ab,kw OR (proportion):ti,ab,kw OR (probability):ti,ab,kw（234155）

#14 #10 OR #11 OR #12 OR #13（883766）

#15 #5 AND #9 AND #14（751）

**5.embase（3752）**

#1 'breast cancer'/exp （558,581）

#2 'breast carcinoma'/exp （108,111）

#3 'breast tumor'/exp （653,676）

#4 'breast cancer':ab,ti （467,625）

#5 'breast neoplasm':ab,ti （463）

#6 'breast carcinoma':ab,ti （34,762）

#7 'breast tumor':ab,ti （16,395）

#8 (breast* NEAR/5 neoplas*):ab,ti （5,689）

#9 (breast* NEAR/5 cancer*):ab,ti （513,610）

#10 (breast* NEAR/5 carcin*):ab,ti （70,237）

#11 (breast* NEAR/5 tumo*):ab,ti （81,816）

#12 (breast* NEAR/5 metasta*):ab,ti （69,243）

#13 (breast* NEAR/5 malig*):ab,ti （22,959）

#14 'mammary cancer*':ab,ti （4,631）

#15 'mammary carcin*':ab,ti （12,965）

#16 'mammary neoplas*':ab,ti （670）

#17 #1 OR #2 OR #3 OR #4 OR #5 OR #6 OR #7 OR #8 OR #9 OR #10 OR #11

OR #12 OR #13 OR #14 OR #15 OR #16 （737,008）

#18 'emotional stress'/exp （26,473）

#19 'distress thermometer'/exp （228）

#20 'psychological distress':ab,ti （32,897）

#21 'psychiatric distress':ab,ti （653）

#22 'emotional distress':ab,ti （11,135）

#23 'mental distress':ab,ti （3,077）

#24 'distress thermometer':ab,ti （2,209）

#25 'emotional stress':ab,ti （7,280）

#26 'distress symptom':ab,ti （227）

#27 distress:ab,ti （201,969）

#28 #18 OR #19 OR #20 OR #21 OR #22 OR #23 OR #24 OR #25 OR #26 OR #27 （223,317）

#29 'risk factor'/exp （1,282,771）

#30 'risk factor*':ab,ti （1,070,980）

#31 risk*:ab,ti （4,009,187）

#32 predictor:ab,ti （358,370）

#33 'predictive factor':ab,ti （17,024）

#34 'influence factor':ab,ti （507）

#35 correlat*:ab,ti （2,908,296）

#36 predict*:ab,ti （2,637,561）

#37 prevalence:ab,ti （1103595）

#38 incidence:ab,ti （1306166）

#39 incident:ab,ti （135386）

#40 epidemiology:ab,ti （234195）

#41 rate:ab,ti （3390890）

#42 frequency:ab,ti （1263048）

#43 occurrence:ab,ti （564549）

#44 morbidity:ab,ti （662026）

#45 proportion:ab,ti （675331）

#46 probability:ab,ti （297667）

#47 #29 OR #30 OR #31 OR #32 OR #33 OR #34 OR #35 OR #36 OR #37 OR #38 OR #39

OR #40 OR #41 OR #42 OR #43 OR #44 OR #45 OR #46

#48 #17 AND #28 AND #47（3752）
