# Supplementary material for: Incidence and risk factors for psychological distress in adult female patients with breast cancer: a systematic review and meta-analysis
Source: Front Psychiatry. 2024 Mar 13;15:1309702. doi: 10.3389/fpsyt.2024.1309702 (PMC10965559; doi:10.3389/fpsyt.2024.1309702)
Supplement: Supplementary file 5 [file Table_2.docx]

| **Supplementary Table 2 Risk of bias for** **Cross-sectional studies.** | | | | | | | | | | | | | |
| --- | --- | --- | --- | --- | --- | --- | --- | --- | --- | --- | --- | --- | --- |
| Authors | Years | Item 1 | Item 2 | Item 3 | Item 4 | Item 5 | Item 6 | Item 7 | Item 8 | Item 9 | Item 10 | Item 11 | Risk of bias |
| Hegel et al.,2006 | 2006 | 1 | 1 | 1 | 1 | 0 | 0 | 1 | 0 | 0 | 1 | 0 | 6 |
| Hegel et al..2008 | 2008 | 1 | 1 | 1 | 1 | 0 | 1 | 1 | 0 | 1 | 1 | 0 | 8 |
| Luutonen et al.,2011 | 2011 | 1 | 1 | 1 | 1 | 1 | 0 | 1 | 1 | 0 | 1 | 0 | 8 |
| Bidstrup et al.,2012 | 2012 | 1 | 1 | 1 | 1 | 0 | 0 | 1 | 0 | 1 | 1 | 0 | 7 |
| Mosher and Duhamel, 2012 | 2012 | 1 | 1 | 0 | 0 | 1 | 1 | 1 | 1 | 0 | 1 | 0 | 7 |
| Mertz et al,2012. | 2012 | 1 | 1 | 0 | 0 | 1 | 0 | 0 | 1 | 1 | 1 | 0 | 6 |
| Head et al.,2012 | 2012 | 1 | 1 | 1 | 1 | 1 | 1 | 1 | 0 | 0 | 1 | 0 | 8 |
| Agarwal et al.,2013 | 2013 | 1 | 1 | 1 | 1 | 0 | 0 | 1 | 1 | 0 | 1 | 0 | 7 |
| Ploos et al.,2013 | 2013 | 1 | 1 | 0 | 0 | 1 | 1 | 1 | 1 | 0 | 1 | 0 | 7 |
| Schmid-Büchi et al.,2013 | 2013 | 1 | 1 | 1 | 1 | 1 | 0 | 1 | 0 | 0 | 1 | 0 | 7 |
| McFarland et al.,2016 | 2016 | 1 | 1 | 1 | 1 | 1 | 0 | 0 | 0 | 0 | 1 | 0 | 6 |
| Ploos et al.,2016 | 2016 | 1 | 1 | 1 | 1 | 1 | 1 | 1 | 1 | 0 | 1 | 0 | 9 |
| Xue et al.,2016 | 2016 | 1 | 1 | 1 | 1 | 0 | 0 | 0 | 0 | 0 | 1 | 0 | 5 |
| Berhili et al.,2017 | 2017 | 1 | 1 | 1 | 1 | 1 | 1 | 0 | 0 | 0 | 0 | 0 | 6 |
| Li et al.,2018 | 2018 | 1 | 1 | 1 | 1 | 0 | 0 | 1 | 0 | 0 | 1 | 0 | 6 |
| Robbeson et al.,2019 | 2019 | 1 | 1 | 1 | 1 | 0 | 1 | 1 | 0 | 0 | 0 | 0 | 6 |
| Cormio et al.,2019 | 2019 | 1 | 1 | 1 | 1 | 0 | 0 | 0 | 1 | 0 | 1 | 0 | 6 |
| Wang et al.,2019 | 2019 | 1 | 1 | 1 | 1 | 1 | 1 | 0 | 0 | 0 | 1 | 0 | 7 |
| Wan et al.,2019 | 2019 | 1 | 1 | 1 | 1 | 0 | 1 | 0 | 0 | 0 | 1 | 0 | 6 |
| Yang et al.,2019 | 2019 | 1 | 1 | 1 | 1 | 0 | 1 | 0 | 0 | 1 | 1 | 0 | 7 |
| Civilotti et al.,2020 | 2020 | 1 | 1 | 0 | 1 | 0 | 0 | 1 | 1 | 0 | 1 | 0 | 6 |
| Sun et al.,2020 | 2020 | 1 | 1 | 1 | 1 | 1 | 1 | 1 | 0 | 0 | 1 | 0 | 8 |
| Budisavljevic et al.,2021 | 2021 | 1 | 1 | 1 | 1 | 1 | 0 | 1 | 1 | 0 | 1 | 0 | 8 |
| Zhao et al.,2022 | 2022 | 1 | 1 | 1 | 1 | 1 | 1 | 1 | 0 | 0 | 1 | 0 | 8 |
| Wang et al.,2022 | 2022 | 1 | 1 | 1 | 1 | 0 | 1 | 1 | 0 | 0 | 1 | 0 | 7 |
| Pang et al..2023 | 2023 | 1 | 1 | 1 | 1 | 0 | 0 | 1 | 1 | 0 | 1 | 0 | 7 |
| Item 1: source of information defined; Item 2: clear criteria for exposed and exposed subjects; Item 3: time period for identifying patients indicated; Item 4: whether or not subjects were consecutive if not population-based indicated; Item 5: whether subjective components of study were masked to other aspects of the status of the participants indicated; Item 6: quality assurance for assessments undertaken; Item 7: patient exclusions from analysis explained; Item 8: confounding assessment or/and control described; Item 9: missing data handling explained; Item 10: response rates and completeness of data collection indicated; Item 11: expected follow-up clarified. | | | | | | | | | | | | | |
