# Supplementary material for: Incidence and risk factors for psychological distress in adult female patients with breast cancer: a systematic review and meta-analysis
Source: Front Psychiatry. 2024 Mar 13;15:1309702. doi: 10.3389/fpsyt.2024.1309702 (PMC10965559; doi:10.3389/fpsyt.2024.1309702)
Supplement: Supplementary file 6 [file Table_3.docx]

| **Supplementary Table3 Risk of bias for Cohort studies.** | | | | | | | | | | | | |
| --- | --- | --- | --- | --- | --- | --- | --- | --- | --- | --- | --- | --- |
| Authors | Years | Selection | | | |  | Comparability |  | Outcome | | | Risk of bias |
|  |  | Representativeness of the exposed cohort | Selection of the non exposed cohort | Ascertainment of exposure | Demonstration that outcome of interest was not Present at start of study |  | Comparability of cohorts on the basis of the design or analysis |  | Assessment of outcome | Was follow-up long enough for outcomes to occur | Adequacy of follow up of cohorts |  |
| Mejdahl et al.,2015 | 2015 | 1 | 1 | 1 | 1 |  | 0 |  | 1 | 0 | 1 | 6 |
| Lo-Fo-Wong et al., 2016 | 2016 | 1 | 1 | 1 | 1 |  | 2 |  | 1 | 1 | 1 | 9 |
| Jørgense et al.,2016 | 2016 | 1 | 1 | 1 | 1 |  | 2 |  | 1 | 1 | 1 | 9 |
| Park et al.,2017 | 2017 | 1 | 1 | 1 | 1 |  | 2 |  | 1 | 1 | 1 | 9 |
| Mertz et al.,2017 | 2017 | 1 | 1 | 1 | 1 |  | 0 |  | 1 | 1 | 1 | 7 |
| NG et al.,2017 | 2017 | 1 | 1 | 1 | 1 |  | 1 |  | 1 | 1 | 0 | 7 |
| Acquati and Kayser., 2017 | 2017 | 1 | 1 | 1 | 0 |  | 1 |  | 1 | 0 | 0 | 5 |
| Shen et al.,2018 | 2018 | 1 | 1 | 1 | 0 |  | 1 |  | 1 | 1 | 1 | 7 |
| Zhang et al.,2018 | 2018 | 1 | 1 | 1 | 0 |  | 0 |  | 1 | 1 | 1 | 6 |
| Ciambella et al.,2019 | 2019 | 1 | 1 | 1 | 0 |  | 2 |  | 1 | 0 | 0 | 6 |
| Fayanju et al.,2020 | 2020 | 1 | 1 | 1 | 0 |  | 2 |  | 1 | 0 | 1 | 7 |
| de Boer et al.,2020 | 2020 | 1 | 1 | 1 | 1 |  | 0 |  | 1 | 1 | 1 | 7 |
| Admiraal et al.,2020 | 2020 | 1 | 1 | 1 | 1 |  | 0 |  | 1 | 1 | 1 | 7 |
| Li et al.,2020 | 2020 | 1 | 1 | 1 | 1 |  | 2 |  | 1 | 1 | 1 | 9 |
| Wang et al.,2021 | 2021 | 1 | 1 | 1 | 1 |  | 1 |  | 1 | 1 | 0 | 7 |
| Tu et al.,2022 | 2022 | 1 | 1 | 0 | 0 |  | 1 |  | 1 | 0 | 1 | 5 |
| Taurisano et al.,2022 | 2022 | 1 | 1 | 1 | 0 |  | 1 |  | 1 | 0 | 1 | 6 |
| Lv et al.,2022 | 2022 | 1 | 1 | 1 | 1 |  | 1 |  | 1 | 0 | 1 | 7 |
| Liu et al.,2022 | 2022 | 1 | 1 | 1 | 1 |  | 1 |  | 1 | 1 | 0 | 7 |
| Liu et al.,2022 | 2022 | 1 | 1 | 0 | 0 |  | 1 |  | 1 | 0 | 1 | 5 |
| Lim et al.,2023 | 2023 | 1 | 1 | 1 | 1 |  | 1 |  | 1 | 0 | 1 | 7 |
| Hass et al.,2023 | 2023 | 1 | 1 | 0 | 1 |  | 1 |  | 1 | 0 | 0 | 5 |
